# Astragaloside IV improves renal function and alleviates renal damage and inflammation in rats with chronic glomerulonephritis

**DOI:** 10.55730/1300-0152.2641

**Published:** 2022-12-09

**Authors:** Dong ZHANG, ZongYing LI, Yuan GAO, HaiLing SUN

**Affiliations:** 1 The First Department of Nephrology, Cangzhou Central Hospital, Hebei Province, China; 2 Department of Hematology, Cangzhou Central Hospital, Hebei Province, China

**Keywords:** Astragaloside IV, miR-181d-5p, CSF1, chronic glomerulonephritis, kidney function, inflammation

## Abstract

From *Astragalus membranaceus* (Fisch.) Bge.var. mongholicus (Bge.) Hsiao, astragaloside IV (AS-IV), a saponin can be purified and is considered traditional Chinese medicine. The purpose of this study was to evaluate the AS-IV-mediated mechanism on chronic glomerulonephritis (CGN). A cationic bovine serum albumin-induced CGN rat model was established and 10, 15, or 20 mg/kg of AS-IV was administered to measure renal function and inflammatory infiltration. Influences of AS-IV on proliferation, cell cycle, and inflammation of LPS-induced rat mesangial cells (RMCs) were determined. The results demonstrated that AS-IV alleviated renal dysfunction, renal lesions, and inflammation in CGN rats. AS-IV prolonged the G0–G1 phase, shortened the S phase, and inhibited cell proliferation and inflammation in RMCs. AS-IV can promote miR-181d-5p expression to inhibit CSF1. miR-181d-5p promotion or CSF1 suppression could further enhance the therapeutic role of AS-IV in CGN rats, while miR-181d-5p silencing or CSF1 overexpression abolished the effect of AS-IV. In conclusion, AS-IV by mediating the miR-181d-5p/CSF1 axis protects against CGN.

## 1. Introduction

The prevalence and disease burden of chronic kidney disease (CKD) has been increasing for decades. Global CKD cases are estimated to exceed 697 million in 2019 (Liang et al., 2022). Belonging to CKD, chronic glomerulonephritis (CGN) is a common cause of end-stage renal disease in patients with CKD (Gao et al., 2022). In clinical practice, drug therapy for CGN has been developed (Fernandez-Juárez et al., 2006; Rovin et al., 2021), but long-term drug use has several side effects, such as muscle wasting, central obesity, hypertension, high cholesterol, and renal dysfunction (Kou et al., 2014; Gao et al., 2016). Therefore, it is requested to find new therapeutic drugs to solve the problem of clinical medication.

It has been confirmed that traditional Chinese medicine has some pharmacological effects, but few side effects (Rodrigues et al., 2016). As an active component of astragalus, astragaloside IV (AS-IV) has multiple pharmacological effects, including antioxidant (Mei et al., 2015; Li et al., 2018), antiinflammatory (Zhou et al., 2017; Zhang et al., 2019), antitumor (Li et al., 2022), and hepatoprotective functions (Zhang et al., 2020). A series of studies have demonstrated the efficacy of AS-IV for kidney diseases, such as obstructive nephropathy (Meng et al., 2011), membranous nephropathy (Zheng et al., 2012), and childhood IgA nephropathy (Liu et al., 2021). Recently, AS-IV has been described to have a renoprotective effect on CGN (Lu et al., 2020). However, current studies have not fully elucidated the underlying mechanism of AS-IV in the treatment of CGN. Therefore, an in-depth study of the regulatory mechanism of AS-IV on CGN progression is still required.

It has been studied that miRNAs mediate up to 30% of genes encoding human proteins (Zhao et al., 2019a). Numerous papers published recently have pointed out that miRNAs take part in the pathogenesis and progression of CKD (Lorenzen et al., 2011; Liu et al., 2018; Peters et al., 2020; Yildirim et al., 2021), including CGN (Kamyshova and Bobkova, 2017; Zhu et al., 2022). miR-181d-5p is a multifunctional miRNA that mediates inflammation by controlling key signaling pathways such as NF-κB signaling (Sun et al., 2012), as well as targets related to immune cell homeostasis (Xue et al., 2011). Recently, it is believed that miR-181d-5p suppresses inflammation and improves renal function in renal injury (Zhang et al., 2020). As previous studies have found that AS-IV has multiple targets, it can control different regulatory pathways by altering miRNAs, thereby participating in the treatment of diseases (Li et al., 2022; Wang et al., 2022a). Cytokines are small proteins that bind receptors on the surface of cell membranes and are involved in promoting cell growth and regulating immune responses, as well as inflammatory responses. Colony-stimulating factor 1 (CSF1) is a cytokine that mainly acts on mononuclear macrophage lines to mediate cell proliferation, differentiation, and function (Hume et al., 2016). CSF1 is involved in the pathogenesis of lupus nephritis in NZBWF1 mice (Li et al., 2021). Therefore, the study hypothesized that miR-181d-5p and CSF1 were also involved in the process of AS-IV improving renal function in CGN rats. A model of C-BSA-induced CGN was established in the study, which is completely similar to human CGN and is considered to be a classical immune complex-induced model of CGN. In addition, inflammation and proliferation of RMCs were induced by LPS to explore the possible mechanism of AS-IV in abnormal proliferation and inflammation of CGN and to determine the relationship between AS-IV and miR-181d-5p/CSF1 axis. This study may provide a theoretical basis and data support for the discovery of CGN therapeutic drugs.

## 2. Methods

### 2.1. Cationic bovine serum albumin (C-BSA)-induced CGN rat model

BSA was provided by Sigma (Lot: #WXBC1232V), and C-BSA was prepared with reference to the Border method (Border et al., 1982). Adult male Sprague–Dawley rats, weighing 180–220 g, were purchased from Chengdu Dossy Experimental Animals Co., Ltd. (2020-034). All animal experimental procedures were approved by the Animal Ethics Committee of Cangzhou Central Hospital (Approval Number: Cz20180311). After 1 week of adaptive feeding, except for the rats in the sham group, the other rats were given C-BSA for 4 weeks to establish the CGN model (Wu et al., 2016). C-BSA was administered at a dose of 1.0 mg on the first 3 days, 1.5 mg on the 4th and 5th days, and 2 mg on the last 2 days; the rats were then administered 2.5 mg of C-BSA intraperitoneally daily for 3 weeks. Twenty-four–hour urinary protein was detected to ensure successful model establishment.

### 2.2. Lentiviral vector construction and injection

The lentiviral vector pLV-CMV containing the cloned sh-CSF1, oe-CSF1, and miR-181d-5p mimic/miR-181d-5p inhibitor were transfected into HEK293T cells with helper vectors pSPAX2 and pMD2G to produce lentivirus (3 × 10^8^ TU/ml).

As shown in Table 1, the rats were randomly divided into 13 groups (6 rats in each group): sham group (no treatment), CGN group, 10 mg/kg group (intravenous injection of 10 mg/kg AS-IV in rats), 15 mg/kg group (intravenous injection of 15 mg/kg AS-IV in rats), 20 mg/kg group (intravenous injection of 20 mg/kg AS-IV in rats), AS-IV + mimic NC group (intravenous injection of 20 mg/kg AS-IV and mimic NC lentivirus in rats), AS-IV + miR-181d-5p mimic group (intravenous injection of 20 mg/kg AS-IV and miR-181d-5p mimic lentivirus in rats), AS-IV + inhibitor NC group (intravenous injection of 20 mg/kg AS-IV and inhibitor NC lentivirus in rats), AS-IV + miR-181d-5p inhibitor group (intravenous injection of 20 mg/kg AS-IV and miR-181d-5p inhibitor lentivirus in rats), AS-IV + sh-NC group (intravenous injection of 20 mg/kg AS-IV and sh-NC lentivirus in rats), AS-IV + sh-CSF1 group (intravenous injection of 20 mg/kg AS-IV and sh-CSF1 lentivirus in rats), AS-IV + oe-NC group (intravenous injection of 20 mg/kg AS-IV and oe-NC lentivirus in rats), and AS-IV + oe-CSF1 group (intravenous injection of 20 mg/kg AS-IV and oe-CSF1 lentivirus in rats). AS-IV > 98% purity was provided by Chengdu Herbpurify Co., Ltd.

### 2.3. Blood collection and kidney tissue resection

After 4 weeks, the rats were anesthetized with isoflurane inhalation, 5 mL of blood was collected from the abdominal aorta, and serum was obtained after centrifugation at 3500 rpm. All kidney tissues were resected from euthanized rats, of which a part was fixed with 10% neutral formalin for histological staining, and the other was frozen in liquid nitrogen (Liang et al., 2014).

### 2.4. Detection of BUN and SCr

Serum BUN and SCr levels were determined using the Hitachi Model 7100 Automatic Analyzer.

### 2.5. HE staining

Kidney tissue samples were dehydrated in graded alcohol and embedded in paraffin for preparation of 5-μm-thick slides for dyeing with hematoxylin (CTS-1099, MXBio, Fuzhou, China) and 0.5% eosin (71,014,544, Sinopharm). After being fixed with neutral glue (G8590; MAIRUI, Shanghai, China), the sections were observed under a microscope (Olympus) in 3 fields of view (Wu et al., 2014).

### 2.6. PAS staining

Paraffin-embedded kidney tissue slides (5 μm) were stained with 10 g/L periodate solution (10,450 60–9, Nanjing Reagent, Nanjing, China), incubated with Schiff solution (DG0005, Leagene Biotech, Beijing, China), combined with hematoxylin (CTS-1099, MXBio), and observed under a microscope (Olympus) in 3 fields of view (Wang et al., 2022b).

### 2.7. Immunohistochemistry

Paraffin-embedded kidney tissue slides (5 μm) were dewaxed with xylene and graded alcohols. After 3% hydrogen peroxide-based inactivation of endogenous peroxidase, slides were combined with rabbit anti-CD68 antibody (1:100, Aobosen, Beijing) and then with polyperoxidase-anti-mouse/rabbit IgG. Followed by DAB development, hematoxylin counterstaining was done, and slides were imaged under a microscope (Olympus) in 3 fields of view.

### 2.8. Cell culture and AS-IV pretreatment

Rat mesangial cells (RMCs; China Center for Type Culture Collection) were maintained in DMEM (Gibco) containing 10% fetal bovine serum (Gibco) which was renewed every 3 days until RMCs were confluent. RMCs were treated with 30 μg/mL LPS (Sigma-Aldrich, St. Louis, MO, USA) for 12 h and pretreated with 10, 20, or 40 μg/mL AS-IV for 24 h (Lu et al., 2020).

### 2.9. Cell transfection

miR-181d-5p inhibitor, inhibitor NC, oe-CSF1, and oe-NC were synthesized by GenePharma (Shanghai, China). Cell transfection was performed using Lipofectamine 2000 (Invitrogen). In brief, RMCs (passages ≤ 16) were seeded on XF96 Seahorse plates at a density of 7000 cells/well and cultured overnight in normal glucose DMEM (GIBCO/ThermoFisher Scientific) with 10% fetal bovine serum (GIBCO/ThermoFisher Scientific). The next day, miR-181d-5p inhibitor, inhibitor NC, oe-CSF1, and oe-NC (40 nM) were transfected into RMCs for 7 h using Lipofectamine 2000 (Lu et al., 2020).

### 2.10. Cell proliferation assay

RMC proliferation was assessed according to the instructions provided by Cell Counting Kit-8 (Bioss, Beijing, China). Briefly, RMCs were collected and transferred into 96-well microplates, incubated with 100 μL of CCK8 solution for 4 h, and then the absorbance at 450 nm was recorded.

### 2.11. Cell cycle analysis

RMCs were seeded in 6-well plates at 1.0 × 10^5^/well and incubated in fetal bovine serum-free DMEM for 12 h. RMCs were then trypsinized, suspended, and fixed in 75% ethanol, and stained with propidium iodide (50 μg/mL; Sigma, USA) and RNaseA. Cellular phases were determined on a BD flow cytometer (BD company) using Cell FIT 2.01.2 (BD company).

### 2.12. ELISA

ELISA kits (Beyotime) were utilized to detect IL-6 and TNF-α in renal tissue and RMCs supernatant.

### 2.13. RT-qPCR

Total RNA was extracted from tissues or cells using TRIzol reagent (Invitrogen) and measured with NanoDrop 2000 (Thermo) according to the concentration and purity of total RNA, and reverse transcription reaction was performed to generate cDNA using the Prime Script RT Reagent Kit (Takara). PCR was performed on Step One PlusTM System (Applied Biosystems). The thermocycling conditions were as follows: Predenaturation at 95 °C for 1 min, 40 cycles at 95 °C for 20 s, and 60 °C for 1 min. The relative expression of each target gene was calculated by the 2^−^^ΔΔ^^Ct^ method. Table 2 presents PCR primers.

### 2.14. Western blot

Based on RIPA lysis buffer (Thermo Fisher Scientific), the extraction of protein was implemented, and the products were subjected to BCA method-based quantification (Beyotime). A total of 30 μg of protein was separated on 12% SDS-PAGE, transferred to PVDF membrane (EMD Millipore), blocked with 5% nonfat milk, and then blocked with primary antibodies CSF1 (3152, 1:1000, Cell Signaling Technology) and GAPDH (ab8245, 1:1000, Abcam). Afterward, goat antirabbit IgG (ab205718; 1:2000; Abcam) was added, and ECL reagent (Cell Signaling Technology) was supplemented to develop protein bands, of which the gray values were assessed using ImageJ 5.0 (Bio-Rad Laboratories).

### 2.15. Dual-luciferase reporter gene assay

Wild-type plasmid CSF1-WT (containing the binding site of miR-181d-5p) and mutant plasmid CSF1-MUT (nucleotides were mutated at the binding site) were constructed. miR-181d-5p mimic or mimic NC (GenePharma) were transfected into RMCs with CSF1-WT or CSF1-MUT according to the instructions of Lipofectamine 2000 (Invitrogen). Relative luciferase activity was calculated according to a dual-luciferase assay kit (Promega).

### 2.16. Statistical analysis

Statistical analysis was performed using SPSS 25.0. All data are presented as mean ± standard deviation. Differences were compared using t-test or one-way analysis of variance. p < 0.05 indicates a statistically significant difference.

## 3. Results

### 3.1. AS-IV can improve renal function and alleviate pathological changes in CGN rats

**Figure 1:**
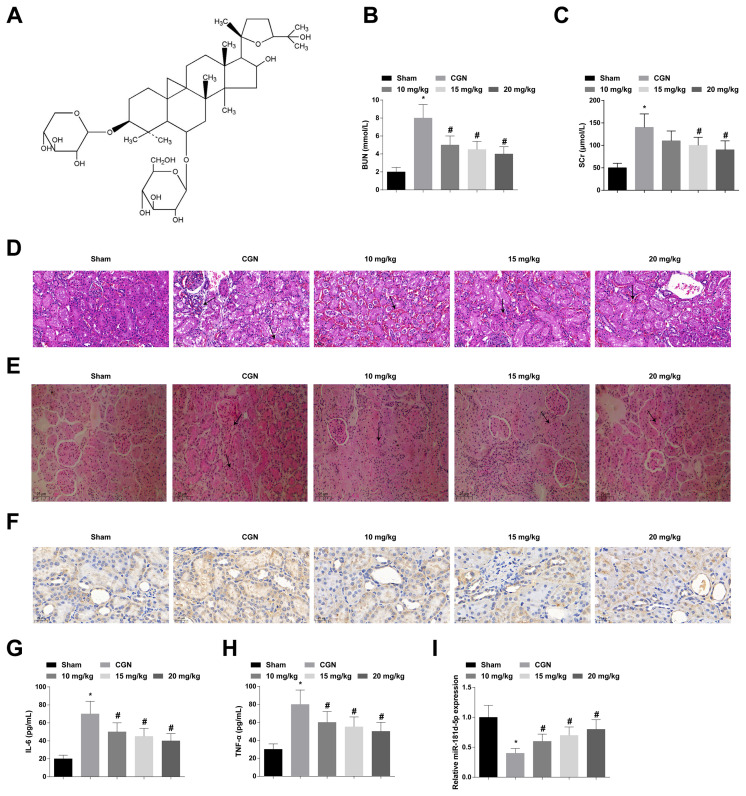
AS-IV can improve renal function and alleviate pathological changes in CGN rats. A: chemical structure of AS-IV; B–C: Automatic biochemical analyzer to determine serum BUN and SCr levels; D–E: HE staining and PAS staining to observe renal histopathologic changes, with black arrows indicating inflammatory infiltration and matrix expansion; F: Immunohistochemistry to measure CD68; G–H: ELASA to analyze IL-6 and TNF-α contents; I: miR-181d-5p expression; values were expressed as mean ± standard deviation (n = 6). * p < 0.05 *vs*. sham; # p < 0.05 *vs*. CGN.

The chemical structure of AS-IV is shown in Figure 1A. To explore the potential regulatory mechanism of AS-IV in CGN, a CGN rat model was established and given 10, 15, or 20 mg/kg of AS-IV. The results revealed that CGN rats exhibited high levels of SCr and BUN, while AS-IV decreased SCr and BUN levels dose-dependently (Figures 1B and 1C). In addition, 24-h urinary protein increased in CGN rats, while AS-IV decreased the 24-h urinary protein in a dose-dependent manner (Figure S1A). The pathological changes of renal tissue of CGN rats were detected by HE staining and PAS staining, showing that the glomeruli and renal tubules of normal rats had a transparent capsule, while the CGN rats had severe pathological damage, mainly manifested as obvious inflammatory cell infiltration, granular degeneration, glomerulus swelling, and marked hyperplasia of the mesangial matrix. Pathological changes were alleviated after AS-IV treatment, and the therapeutic effect of AS-IV was dose-dependent (Figures 1D and 1E). To measure the inflammatory changes in CGN rats, immunohistochemistry was performed to detect CD68 and ELISA to determine inflammatory factors. Immunohistochemical results found CD68 mainly in the cytoplasm of glomeruli and tubulointerstitium, and that the infection of macrophages in the kidney tissue of CGN rats was increased, while AS-IV was a dose-dependent way to reduce the inflammatory response (Figure 1F). ELISA results manifested that IL-6 and TNF-α contents were augmented in CGN rats, while AS-IV decreased IL-6 and TNF-α contents dose-dependently (Figures 1G and 1H). miR-181d-5p was decreased in CGN rats, while AS-IV upregulated miR-181d-5p dose-dependently (Figure 1I). In conclusion, AS-IV can improve renal function and alleviate pathological changes in CGN rats, and 20 mg/kg AS-IV has the best therapeutic effect. Therefore, 20 mg/kg of AS-IV was selected for subsequent experiments.

### 3.2. miR-181d-5p has a healing effect on CGN rats

**Figure 2:**
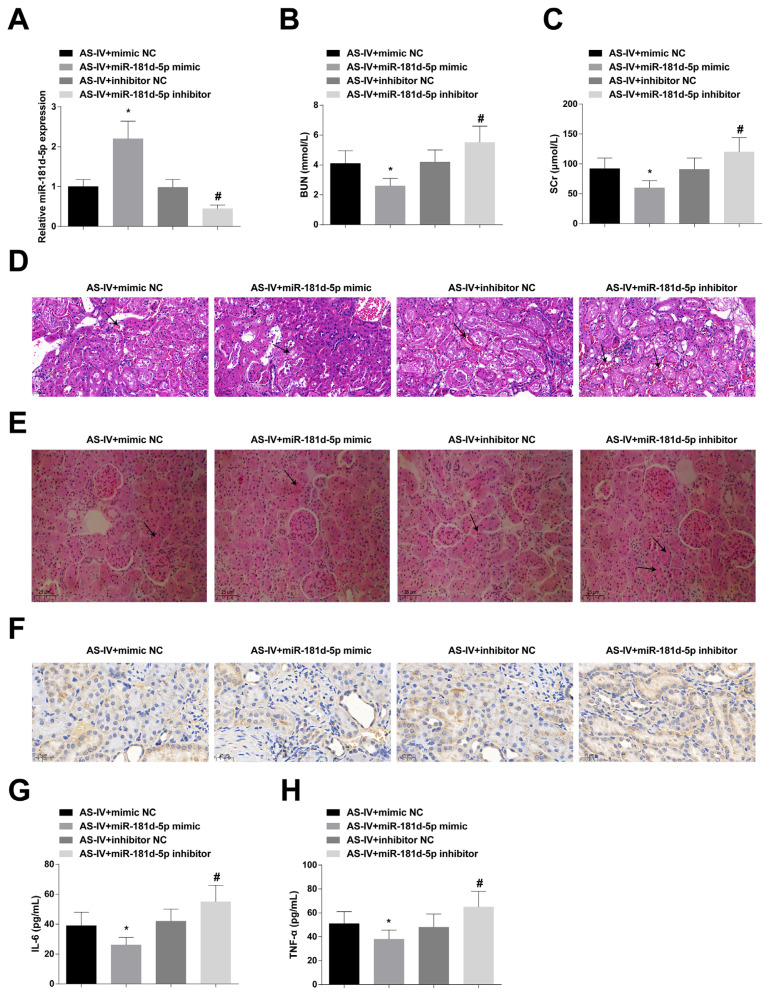
miR-181d-5p has a healing effect on CGN rats. A: RT-qPCR to detect miR-181d-5p expression; B–C: After regulating miR-181d-5p, Automatic biochemical analyzer to determine serum BUN and SCr levels; D–E: After regulating miR-181d-5p, HE staining and PAS staining to observe renal histopathologic changes, with black arrows indicating inflammatory infiltration and matrix expansion; F: After regulating miR-181d-5p, Immunohistochemistry to measure CD68; G–H: After regulating miR-181d-5p, ELASA to analyze IL-6 and TNF-α contents; values were expressed as mean ± standard deviation (n = 6). * p < 0.05 *vs*. AS-IV + mimic NC; # p < 0.05 *vs*. AS-IV + inhibitor NC.

CGN rats were given 20 mg/kg AS-IV and injected with lentivirus that interfered with miR-181d-5p. RT-qPCR results confirmed that miR-181d-5p expression intervention was successful (Figure 2A). SCr and BUN levels were further reduced after overexpressing miR-181d-5p, while inhibiting miR-181d-5p could mitigate AS-IV-mediated SCr and BUN levels (Figures 2B and 2C). Twenty-four–hour urinary protein was further decreased after upregulating miR-181d-5p, while downregulating miR-181d-5p could reverse the effect of AS-IV on 24-h urinary protein (Figure S1B). miR-181d-5p could further alleviate renal tissue pathological damage and inflammatory response, while miR-181d-5p inhibition blocked the improvement effect of AS-IV on renal tissue pathological damage and inflammatory response (Figures 2D–2H).

### 3.3. miR-181d-5p inhibits CSF1 expression

**Figure 3:**
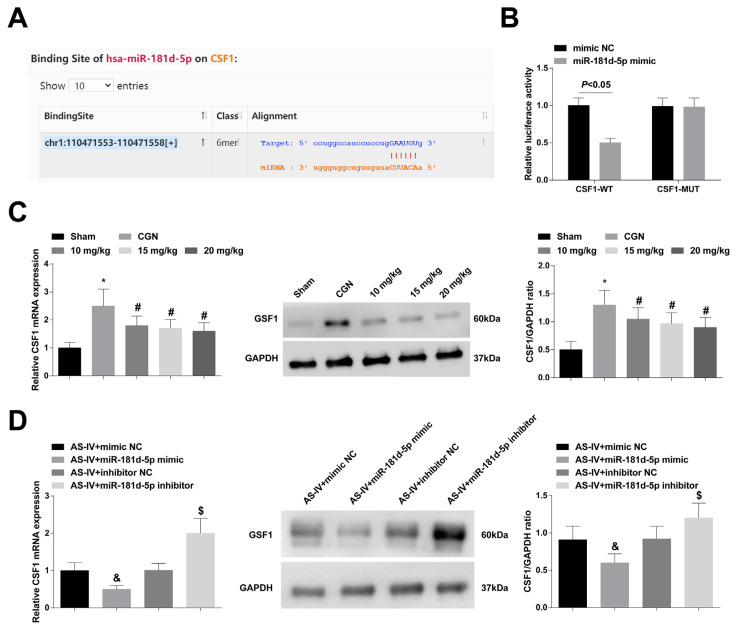
miR-181d-5p inhibits CSF1 expression. A: Bioinformatics website predicted the targeted binding site of miR-181d-5p and CSF1; B: Dual luciferase reporter assay verified the targeting binding of miR-181d-5p to CSF1 (N = 3); C–D: RT-qPCR and Western blot to analyze CSF1 expression in rats (n = 6); values were expressed as mean ± standard deviation. * p < 0.05 *vs*. sham; # p < 0.05 *vs*. CGN; & p < 0.05 *vs*. AS-IV + mimic NC; $ p < 0.05 *vs*. AS-IV + inhibitor NC.

By starbase database (https://starbase.sysu.edu.cn/), CSF1 might be a target gene of miR-181d-5p (Figure 3A). The detection results of luciferase activity showed that miR-downregulated by AS-IV dose-dependently (Figure 3C). 181d-5p mimic decreased firefly luciferase activity of In addition, CSF1 expression was further decreased after CSF1-WT (Figure 3B). In further studies, it was found overexpressing miR-181d-5p, while it was increased after that CSF1 was upregulated in CGN rats, while it was suppressing miR-181d-5p (Figure 3D).

### 3.4. CSF1 inhibition protects against CGN in rats

**Figure 4:**
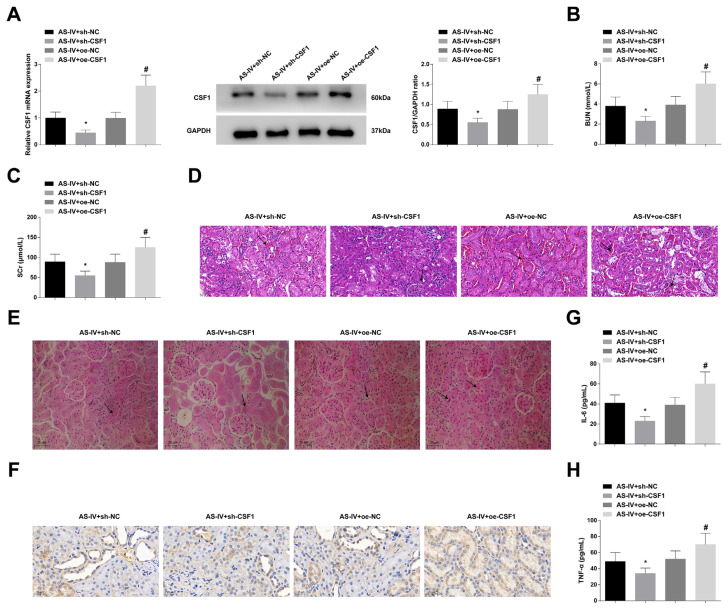
CSF1 inhibition protects against CGN in rats. A: RT-qPCR and Western blot to analyze CSF1 expression; B–C: After regulating CSF1, Automatic biochemical analyzer to determine serum BUN and SCr levels; D–E: After regulating CSF1, HE staining and PAS staining to observe renal histopathologic changes, with black arrows indicating inflammatory infiltration and matrix expansion; F: After regulating CSF1, Immunohistochemistry to measure CD68; G–H: After regulating CSF1, ELASA to analyze IL-6 and TNF-α contents; values were expressed as mean ± standard deviation (n = 6). * p < 0.05 *vs*. AS-IV + sh-NC; # p < 0.05 *vs*. AS-IV + oe-NC.

With treatment with 20 mg/kg of AS-IV, CGN rats were injected with lentivirus that interfered with CSF1 expression. RT-qPCR and Western blot showed that CSF1 expression was successfully intervened (Figure 4A). CSF1 low expression further reduced SCr, BUN, and 24-h urinary protein, alleviated the pathological damage of renal tissue, and attenuated inflammatory response based on AS-IV treatment, while CSF1 promotion worsened the performance of AS-IV on CGN rats (Figures 4B–4H, Figure S1C).

### 3.5. AS-IV can inhibit RMCs proliferation and inflammation

**Figure 5:**
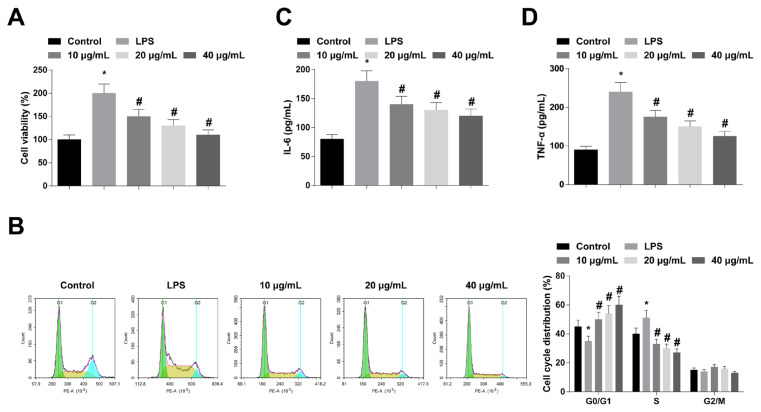
AS-IV can inhibit RMCs proliferation and inflammation. A: CCK-8 to measure RMCs proliferation; B: Flow cytometry to determine RMCs cell cycle; C–D: ELASA to analyze IL-6 and TNF-α contents in RMCs supernatant; values were expressed as mean ± standard deviation (N = 3). * p < 0.05 *vs*. control; # p < 0.05 *vs*. LPS.

To further validate the therapeutic effect of AS-IV on CGN, RMCs were pretreated with AS-IV at 10, 20, and 40 μg/mL, and then cellular inflammation was induced by LPS. CCK-8 results showed that AS-IV inhibited LPS-induced proliferation of RMCs dose-dependently (Figure 5A). Cell cycle detection by flow cytometry implicated that AS-IV prolonged the G0–G1 phase and shortened the S phase dose-dependently in LPS-induced RMCs (Figure 5B). ELISA finding revealed that AS-IV decreased IL-6 and TNF-α in LPS-induced RMCs dose-dependently (Figures 5C and 5D).

### 3.6. miR-181d-5p deficiency or CSF1 induction can aggrandize the performance of AS-IV

**Figure 6:**
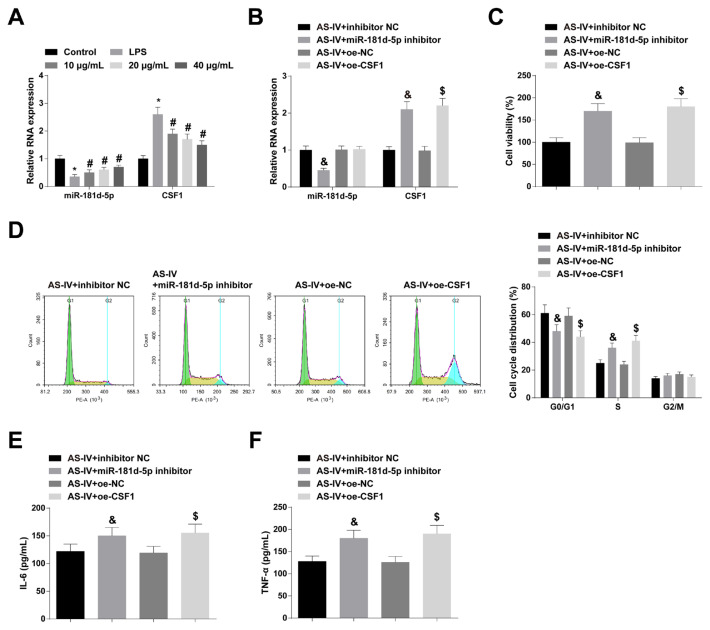
miR-181d-5p deficiency or CSF1 induction can aggrandize the effect of AS-IV. B: RT-qPCR to detect miR-181d-5p and CSF1 expression; C: After downregulating CSF1 or upregulating CSF1, CCK-8 to measure RMCs proliferation; D: After downregulating CSF1 or upregulating CSF1, Flow cytometry to determine RMCs cell cycle; E–F: After downregulating CSF1 or upregulating CSF1, ELASA to analyze IL-6 and TNF-α contents in RMCs supernatant; values were expressed as mean ± standard deviation (N = 3). * p < 0.05 *vs*. Control; # p < 0.05 *vs*. LPS; & P < 0.05 *vs*. AS-IV + inhibitor NC; $ p < 0.05 *vs*. AS-IV+oe-NC.

AS-IV promoted miR-181d-5p expression and inhibited CSF1 expression dose-dependently (Figure 6A). RMCs pretreated with 40 μg/mL of AS-IV were transfecting miR-181d-5p inhibitor or oe-CSF1 to mediate gene expression (Figure 6B). Cellular experiments suggested that miR-181d-5p suppression or CSF1 overexpression reduced AS-IV-mediated performance on RMCs proliferation (Figure 6C), cell cycle (Figure 6D), and inflammatory factor production (Figures 6E and 6F).

## 4. Discussion

CGN is associated with immune-mediated inflammatory disease, frequently occurs during end-stage renal disease, and severely affects patient survival (Ding et al., 2013; Gao et al., 2016). Traditional Chinese medicine has advantages in the treatment of complex diseases (Zhao et al., 2019b), and particularly AS-IV is of clinical significance to treat various renal diseases (Meng et al., 2011; Zheng et al., 2012; Lu et al., 2020; Liu et al., 2021).

The establishment of appropriate models is the basis for simulating diseases. The C-BSA-induced CGN rat model was chosen for this study because it has previously been shown to be similar to human CGN progression (Wu et al., 2016). Studies have reported that immune-mediated inflammation and glomerular injury in the tubulointerstitial compartment are the keys to CGN progression (Zou et al., 2012; Schwalm et al., 2014; Velciov et al., 2016). The present study found that CGN rats showed significant deterioration of renal function, accompanied by massive 24-h urinary protein and abnormal serum BUN and SCr levels. CGN rats showed obvious pathological injury and inflammatory symptoms. Interestingly, AS-IV treatment could improve the biochemical indicators of blood renal function in C-BSA-induced CGN rats, alleviate renal pathological damage, and inhibit the inflammatory response. Common pathologies of proliferative and inflammatory glomerular diseases in humans and experimental animals include mesangial cell proliferation (Cao et al., 2015; Singh et al., 2016; Kurihara and Sakai 2017), which may lead to excessive deposition of extracellular matrix, glomerulosclerosis, and loss of renal function. LPS is considered to be one of the strong stimulators of RMCs, and it can be used as an inducer of glomerular cell viability (Gao et al., 2018). Therefore, LPS was employed to mimic CGN in cells. The present study found that AS-IV could inhibit RMC proliferation and inflammation, which is consistent with previous findings (Wu et al., 2018).

miR-181d-5p is a multifunctional miRNA that mediates inflammation and immune cell homeostasis (Sun et al., 2012) and is a great mediator for inflammation and improves renal function in renal injury (Xue et al., 2011; Zhang et al., 2020). Interestingly, miR-181d-5p was decreased in CGN rats, whereas AS-IV upregulated miR-181d-5p dose-dependently. Given that, it was speculated that AS-IV may improve renal function and alleviate pathological changes in CGN rats by upregulating miR-181d-5p. To further test our hypothesis, CGN rats were injected with 20 mg/kg of AS-IV simultaneously with lentivirus that interfered with miR-181d-5p. As the findings indicate, promoting miR-181d-5p expression could further enhance the therapeutic effect of AS-IV in CGN rats, while declining miR-181d-5p expression could attenuate the therapeutic effect of AS-IV in CGN rats.

CSF1 is a critical modifier of macrophage production, differentiation, and function (Zhang et al., 2021). CSF1 is upregulated in renal tubular epithelial cells in response to renal injury stimuli (Perry and Okusa, 2015) and could mediate inflammatory damage and apoptosis in human mesangial cells in lupus nephritis (Liao et al., 2022). Here, it was measured that CSF1 expression was upregulated in CGN rats, whereas it was downregulated by AS-IV and further decreased after upregulation of miR-181d-5p. Based on this, further functional rescue experiments were carried out, demonstrating that CSF1 inhibition could further enhance the therapeutic effect of AS-IV on CGN rats, while CSF1 did the opposite. Furthermore, in vitro cell experiments further support that AS-IV improves renal function in rats with CGN by regulating the miR-181d-5p/CSF1 axis, and attenuates renal damage and inflammation.

However, a limitation is that our findings are based on cell culture investigations and experimental animal studies without clinical practice. Furthermore, the NF-κB signaling pathway is related to the inflammatory pathogenesis of CGN (Chen et al., 2022), while AS-IV can activate different functional pathways (Zhang et al., 2022). Therefore, the downstream pathways of AS-IV can be further elucidated in the future to enrich our study.

## 5. Conclusion

AS-IV improves renal function in CGN rats by mediating the miR-181d-5p/CSF1 axis and alleviates renal lesions and inflammation. In vitro, it prolongs the G0–G1 phase and shortens the S phase in RMCs, and inhibits cell proliferation and inflammation. AS-IV has the potential to become a clinical drug for the treatment of CGN. AS-IV therapy in the clinic warrants further investigation as an alternative treatment strategy to manage the progression of CGN and end-stage renal disease.

## Supplementary

Figure S1 Detection of 24-h urinary protein in CGN rats.
A–C: 24 h urinary protein of rats; Values were expressed as mean ± standard deviation (n = 6). *p < 0.05 *vs*. sham; # p < 0.05 *vs*. CGN; & p < 0.05 *vs*. AS-IV + mimic NC; $ p < 0.05 *vs*. AS-IV + inhibitor NC; ^ p < 0.05 *vs*. AS-IV + sh-NC; ^ p < 0.05 *vs*. AS-IV + oe-NC.

## Figures and Tables

**Table 1:** Animal groups.

Groups	Treatments
Sham	No treatment
CGN	CGN rat model was induced by C-BSA
10 mg/kg	Intravenous injection of 10 mg/kg AS-IV in CGN rats
15 mg/kg	Intravenous injection of 15 mg/kg AS-IV in CGN rats
20 mg/kg	Intravenous injection of 20 mg/kg AS-IV in CGN rats
AS-IV+mimic NC	Intravenous injection of 20 mg/kg AS-IV and mimic NC lentivirus in CGN rats
AS-IV + miR-181d-5p mimic	Intravenous injection of 20 mg/kg AS-IV and miR-181d-5p mimic lentivirus in CGN rats
AS-IV + inhibitor NC	Intravenous injection of 20 mg/kg AS-IV and miR-181d-5p inhibitor NC lentivirus in CGN rats
AS-IV + miR-181d-5p inhibitor	Intravenous injection of 20 mg/kg AS-IV and miR-181d-5p inhibitor NC lentivirus in CGN rats
AS-IV + sh-NC	Intravenous injection of 20 mg/kg AS-IV and sh-NC lentivirus in CGN rats
AS-IV + sh-CSF1	Intravenous injection of 20 mg/kg AS-IV and sh-CSF1 lentivirus in CGN rats
AS-IV + oe-NC	Intravenous injection of 20 mg/kg AS-IV and oe-NC lentivirus in CGN rats
AS-IV + oe-CSF1	Intravenous injection of 20 mg/kg AS-IV and oe-CSF1 lentivirus in CGN rats

**Table 2:** Primers.

Genes	Primers (5′–3′)	Tm degree
miR-181d-5p	Forward: CGAACATTCATTGTTGTCG	52.3 °C
	Reverse: GCAGGGTCCGAGGTATTC	54.7 °C
CSF1	Forward: TGGCGAGCAGGAGTATCAC	56.4 °C
	Reverse: AGGTCTCCATCTGACTGTCAAT	55.1 °C
U6	Forward: CTCGCTTCGGCAGCACA	59.3 °C
	Reverse: AACGCTTCACGAATTTGCGT	60.8 °C
GAPDH	Forward: GTCGGTGTGAACGGATTTG	56.5 °C
	Reverse: TCCCATTCTCAGCCTTGAC	55.2 °C

Note: miR-181d-5p, microRNA-181d-5p; CSF1, colony-stimulating factor 1; GAPDH, glyceraldehyde-3-phosphate dehydrogenase

## References

[b1-turkjbiol-47-1-61] Border WA Ward HJ Kamil ES Cohen AH 1982 Induction of membranous nephropathy in rabbits by administration of an exogenous cationic antigen The Journal of clinical investigation 69 451 61 10.1172/jci110469 7056856 PMC370995

[b2-turkjbiol-47-1-61] Cao Y Huang X Fan Y Chen X 2015 Protective Effect of Triptolide against Glomerular Mesangial Cell Proliferation and Glomerular Fibrosis in Rats Involves the TGF-β 1/Smad Signaling Pathway Evidence-Based Complementary and Alternative Medicine: eCAM 2015 814089 10.1155/2015/814089 PMC4584226 26451157

[b3-turkjbiol-47-1-61] Chen Q Guo H Hu J Zhao X 2022 Rhein Inhibits NF-κB Signaling Pathway to Alleviate Inflammatory Response and Oxidative Stress of Rats with Chronic Glomerulonephritis Applied Bionics and Biomechanics 2022 9671759 10.1155/2022/9671759 PMC9020916 35465184

[b4-turkjbiol-47-1-61] Ding SY Zheng PD He LQ Hou WG Zou Y 2013 The research on xiaochalhu decoction improving the inflammation of chronic glomerulonephritis patients and relieving the proteinuria Zhongguo Zhong Xi Yi Jie He Za Zhi 33 1 21 6 23596780

[b5-turkjbiol-47-1-61] Fernandez-Juárez G Barrio V de Vinuesa SG Goicoechea M Manuel Praga 2006 Dual blockade of the renin-angiotensin system in the progression of renal disease: the need for more clinical trials Journal of the American Society of Nephrology 17 S250 4 10.1681/asn.2006080922 17130270

[b6-turkjbiol-47-1-61] Gao JR Qin XJ Jiang H Wang T Song JM 2016 The effects of Qi Teng Xiao Zhuo granules, traditional Chinese medicine, on the expression of genes in chronic glomerulonephritis rats Journal of Ethnopharmacology 193 140 49 10.1016/j.jep.2016.08.011 27497640

[b7-turkjbiol-47-1-61] Gao J Wei L Song J Jiang H Gao Y 2018 In vitro and in vivo study of the expression of the Syk/Ras/c-Fos pathway in chronic glomerulonephritis Molecular Medicine Reports 18 3683 90 10.3892/mmr.2018.9355 30106104 PMC6131599

[b8-turkjbiol-47-1-61] Gao J Zhu X Chen H Jiang H Shi M 2022 Long Non-Coding NONRATG0019102 Promotes the Proliferation of Rat Mesangial Cell Line HBZY-1 Through the miR-339-3p/CTNNB1 Axis Frontiers in Genetics 13 834144 10.3389/fgene.2022.834144 35571052 PMC9096093

[b9-turkjbiol-47-1-61] Hume DA Summers KM Rehli M 2016 Transcriptional Regulation and Macrophage Differentiation Microbiology Spectrum 4 10.1128/microbiolspec.MCHD-0024-2015 27337479

[b10-turkjbiol-47-1-61] Kamyshova ES Bobkova IN 2017 MicroRNAs in chronic glomerulonephritis: Promising biomarkers for diagnosis and prognosis estimation Terapevticheskii Arkhiv 89 89 96 10.17116/terarkh201789689-96 28745695

[b11-turkjbiol-47-1-61] Kou J Wu J Yang HT He YN Fang JA 2014 Efficacy and safety of Shenyankangfu tablets for primary glomerulonephritis: study protocol for a randomized controlled trial Trials 15 479 10.1186/1745-6215-15-479 25480673 PMC4289030

[b12-turkjbiol-47-1-61] Kurihara H Sakai T 2017 Cell biology of mesangial cells: the third cell that maintains the glomerular capillary Anatomical Science International 92 173 86 10.1007/s12565-016-0334-1 26910209

[b13-turkjbiol-47-1-61] Li F Cao K Wang M Liu Y Zhang Y 2022 Astragaloside IV exhibits anti-tumor function in gastric cancer via targeting circRNA dihydrolipoamide S-succinyltransferase (circDLST)/miR-489-3p/eukaryotic translation initiation factor 4A1(EIF4A1) pathway Bioengineered 13 10111 22 10.1080/21655979.2022.2063664 35435117 PMC9161858

[b14-turkjbiol-47-1-61] Li L Huang W Wang S Sun K Zhang W 2018 Astragaloside IV Attenuates Acetaminophen-Induced Liver Injuries in Mice by Activating the Nrf2 Signaling Pathway Molecules (Basel Switzerland) 23 10.3390/molecules23082032 30110942 PMC6222748

[b15-turkjbiol-47-1-61] Li S Wang X Zhu X Xue Y Zhang J 2021 miR-1968-5p is involved in the pathogenesis of lupus nephritis of NZBWF1 mice by targeting csf1 Clinical and Experimental Nephrology 25 1173 81 10.1007/s10157-021-02091-y 34231109

[b16-turkjbiol-47-1-61] Liang CL Wu JB Lai JM Ye SF Lin J 2014 Protection Effect of Zhen-Wu-Tang on Adriamycin-Induced Nephrotic Syndrome via Inhibiting Oxidative Lesions and Inflammation Damage Evidence-Based Complementary and Alternative Medicine: eCAM 2014 131604 10.1155/2014/131604 PMC4000650 24812565

[b17-turkjbiol-47-1-61] Liang Z Wang W Yang C Wang Y Shen J 2022 Residential greenness and prevalence of chronic kidney disease: Findings from the China National Survey of Chronic Kidney Disease The Science of the Total Environment 806 150628 10.1016/j.scitotenv.2021.150628 34592294

[b18-turkjbiol-47-1-61] Liao W He XJ Zhang W Chen YL Yang J 2022 MiR-145 participates in the development of lupus nephritis by targeting CSF1 to regulate the JAK/STAT signaling pathway Cytokine 154 155877 10.1016/j.cyto.2022.155877 35468468

[b19-turkjbiol-47-1-61] Liu C Li X Shuai L Dang X Peng F 2021 Astragaloside IV Inhibits Galactose-Deficient IgA1 Secretion via miR-98-5p in Pediatric IgA Nephropathy Frontiers in Pharmacology 12 658236 10.3389/fphar.2021.658236 33935780 PMC8085534

[b20-turkjbiol-47-1-61] Liu L Pang XL Shang WJ Xie HC Wang JX 2018 Over-expressed microRNA-181a reduces glomerular sclerosis and renal tubular epithelial injury in rats with chronic kidney disease via down-regulation of the TLR/NF-κB pathway by binding to CRY1 Molecular Medicine (Cambridge, Mass) 24 49 10.1186/s10020-018-0045-2 30241461 PMC6145098

[b21-turkjbiol-47-1-61] Lorenzen JM Haller H Thum T 2011 MicroRNAs as mediators and therapeutic targets in chronic kidney disease Nature Reviews Nephrology 7 286 94 10.1038/nrneph.2011.26 21423249

[b22-turkjbiol-47-1-61] Lu R Chen J Liu B Lin H Bai L 2020 Protective role of Astragaloside IV in chronic glomerulonephritis by activating autophagy through PI3K/AKT/AS160 pathway Phytotherapy Research: PTR 34 3236 48 10.1002/ptr.6772 32726508

[b23-turkjbiol-47-1-61] Mei M Tang F Lu M He X Wang H 2015 Astragaloside IV attenuates apoptosis of hypertrophic cardiomyocyte through inhibiting oxidative stress and calpain-1 activation Environmental Toxicology and Pharmacology 40 764 73 10.1016/j.etap.2015.09.007 26433482

[b24-turkjbiol-47-1-61] Meng LQ Tang JW Wang Y Zhao JR Shang MY 2011 Astragaloside IV synergizes with ferulic acid to inhibit renal tubulointerstitial fibrosis in rats with obstructive nephropathy British Journal of Pharmacology 162 1805 18 10.1111/j.1476-5381.2011.01206.x 21232035 PMC3081123

[b25-turkjbiol-47-1-61] Perry HM Okusa MD 2015 Driving change: kidney proximal tubule CSF-1 polarizes macrophages Kidney International 88 1219 21 10.1038/ki.2015.324 26649657 PMC4976441

[b26-turkjbiol-47-1-61] Peters LJF Floege J Biessen EAL Jankowski J van der Vorst EPC 2020 MicroRNAs in Chronic Kidney Disease: Four Candidates for Clinical Application International Journal of Molecular Sciences 21 10.3390/ijms21186547 PMC7555601 32906849

[b27-turkjbiol-47-1-61] Rodrigues T Reker D Schneider P Schneider G 2016 Counting on natural products for drug design Nature Chemistry 8 531 41 10.1038/nchem.2479 27219696

[b28-turkjbiol-47-1-61] Rovin BH Adler SG Barratt J Bridoux F Burdge KA 2021 Executive summary of the KDIGO 2021 Guideline for the Management of Glomerular Diseases Kidney International 100 753 79 10.1016/j.kint.2021.05.015 34556300

[b29-turkjbiol-47-1-61] Schwalm S Pfeilschifter J Huwiler A 2014 Targeting the sphingosine kinase/sphingosine 1-phosphate pathway to treat chronic inflammatory kidney diseases Basic & Clinical Pharmacology & Toxicology 114 44 9 10.1111/bcpt.12103 23789924

[b30-turkjbiol-47-1-61] Singh U Rai V Singh R Santosh D Parkash J 2016 Renal Biopsy Findings in Patients with Hypothyroidism: Report of 16 cases Journal of Clinical and Diagnostic Research: JCDR 10 Ec27 9 10.7860/jcdr/2016/19362.8356 PMC5028462 27656449

[b31-turkjbiol-47-1-61] Sun X Icli B Wara AK Belkin N He S 2012 MicroRNA-181b regulates NF-κB-mediated vascular inflammation The Journal of Clinical Investigation 122 1973 90 10.1172/jci61495 22622040 PMC3366408

[b32-turkjbiol-47-1-61] Velciov S Gluhovschi G Timar R Gluhovschi C Petrica L 2016 Urinary enzymatic markers (N-acetyl-beta-D-glucosaminidase) in assessing the tubulointerstitial compartment in chronic glomerulonephritis related to odontogenic foci Wiener Klinische Wochenschrift 128 102 8 10.1007/s00508-015-0841-4 26377174

[b33-turkjbiol-47-1-61] Wang Q Chen W Yang X Song Y Sun X 2022a Inhibition of miRNA-1-Mediated Inflammation and Autophagy by Astragaloside IV Improves Lipopolysaccharide-Induced Cardiac Dysfunction in Rats Journal of Inflammation Research 15 2617 29 10.2147/jir.S362368 35494314 PMC9045596

[b34-turkjbiol-47-1-61] Wang Y Feng F He W Sun L He Q 2022b miR-188-3pabolishes germacrone-mediated podocyte protection in a mouse model of diabetic nephropathy in type I diabetes through triggering mitochondrial injury Bioengineered 13 774 88 10.1080/21655979.2021.2012919 34847832 PMC8805940

[b35-turkjbiol-47-1-61] Wu JB Ye SF Liang CL Li YC Yu YJ 2014 Qi-Dan Fang ameliorates adriamycin-induced nephrotic syndrome rat model by enhancing renal function and inhibiting podocyte injury Journal of Ethnopharmacology 151 1124 32 10.1016/j.jep.2013.12.028 24389029

[b36-turkjbiol-47-1-61] Wu J He Y Luo Y Zhang L Lin H 2018 MiR-145-5p inhibits proliferation and inflammatory responses of RMC through regulating AKT/GSK pathway by targeting CXCL16 Journal of Cellular Physiology 233 3648 59 10.1002/jcp.26228 29030988

[b37-turkjbiol-47-1-61] Wu J Liu B Liang C Ouyang H Lin J 2016 Zhen-wutang attenuates cationic bovine serum albumin-induced inflammatory response in membranous glomerulonephritis rat through inhibiting AGEs/RAGE/NF-κB pathway activation International Immunopharmacology 33 33 41 10.1016/j.intimp.2016.01.008 26851631

[b38-turkjbiol-47-1-61] Xue Q Guo ZY Li W Wen WH Meng YL 2011 Human activated CD4(+) T lymphocytes increase IL-2 expression by downregulating microRNA-181c Molecular Immunology 48 592 9 10.1016/j.molimm.2010.10.021 21112091

[b39-turkjbiol-47-1-61] Yildirim D Bender O Karagoz ZF Helvacioglu F Bilgic MA 2021 Role of autophagy and evaluation the effects of microRNAs 214, 132, 34c and prorenin receptor in a rat model of focal segmental glomerulosclerosis Life Sciences 280 119671 10.1016/j.lfs.2021.119671 34087284

[b40-turkjbiol-47-1-61] Zhang J Wu C Gao L Du G Qin X 2020 Astragaloside IV derived from Astragalus membranaceus: A research review on the pharmacological effects Advances in Pharmacology (San Diego, Calif) 87 89 112 10.1016/bs.apha.2019.08.002 32089240

[b41-turkjbiol-47-1-61] Zhang R Zhang X Xing B Zhao J Zhang P 2019 Astragaloside IV attenuates gestational diabetes mellitus via targeting NLRP3 inflammasome in genetic mice Reproductive Biology and Endocrinology: RB&E 17 77 10.1186/s12958-019-0522-7 31558153 PMC6764134

[b42-turkjbiol-47-1-61] Zhang Y Du M Wang J Liu P 2022 Astragaloside IV Relieves Atherosclerosis and Hepatic Steatosis via MAPK/NF-κB Signaling Pathway in LDLR(-/-) Mice Frontiers in Pharmacology 13 828161 10.3389/fphar.2022.828161 35264962 PMC8899310

[b43-turkjbiol-47-1-61] Zhang YH Chen AL Yu RQ Jia BB Ye DN 2021 miR-296-5p Inhibits the Secretion of Pulmonary Surfactants in Pulmonary Epithelial Cells via the Downregulation of Wnt7b/β-Catenin Signaling BioMed Research International 2021 4051504 10.1155/2021/4051504 33490270 PMC7803427

[b44-turkjbiol-47-1-61] Zhang Y Li C Guan C Zhou B Wang L 2020 MiR-181d-5p Targets KLF6 to Improve Ischemia/Reperfusion-Induced AKI Through Effects on Renal Function Apoptosis, and Inflammation, Frontiers in Physiology 11 510 10.3389/fphys.2020.00510 32581828 PMC7295155

[b45-turkjbiol-47-1-61] Zhao H Ma SX Shang YQ Zhang HQ Su W 2019a microRNAs in chronic kidney disease. Clinica Chimica Acta International Journal of Clinical Chemistry 491 59 65 10.1016/j.cca.2019.01.008 30639583

[b46-turkjbiol-47-1-61] Zhao J Chan YC He B Duan TT Yu ZL 2019b A patent herbal drug Yi-Shen-Hua-Shi granule ameliorates C-BSA-induced chronic glomerulonephritis and inhabits TGFβ signaling in rats Journal of Ethnopharmacology 236 258 62 10.1016/j.jep.2019.02.044 30836175

[b47-turkjbiol-47-1-61] Zheng R Deng Y Chen Y Fan J Zhang M 2012 Astragaloside IV attenuates complement membranous attack complex induced podocyte injury through the MAPK pathway Phytotherapy Research: PTR 26 892 8 10.1002/ptr.3656 22086717

[b48-turkjbiol-47-1-61] Zhou X Sun X Gong X Yang Y Chen C 2017 Astragaloside IV from Astragalus membranaceus ameliorates renal interstitial fibrosis by inhibiting inflammation via TLR4/NF-κB in vivo and in vitro International Immunopharmacology 42 18 24 10.1016/j.intimp.2016.11.006 27855303

[b49-turkjbiol-47-1-61] Zhu X Tang L Mao J Hameed Y Zhang J 2022 Decoding the Mechanism behind the Pathogenesis of the Focal Segmental Glomerulosclerosis Computational and Mathematical Methods in Medicine 2022 1941038 10.1155/2022/1941038 PMC9175094 35693262

[b50-turkjbiol-47-1-61] Zou L Wang W Xu Z Zhang N Jiang T 2012 Aldose reductase regulates platelet-derived growth factor-induced proliferation through mediating cell cycle progression in rat mesangial cells International Journal of Molecular Medicine 30 409 16 10.3892/ijmm.2012.997 22614447

