# Supplementary figures and images for: Astragaloside IV improves renal function and alleviates renal damage and inflammation in rats with chronic glomerulonephritis
Source: Turk J Biol. 2022 Dec 9;47(1):61–73. doi: 10.55730/1300-0152.2641 (PMC10387845; doi:10.55730/1300-0152.2641)

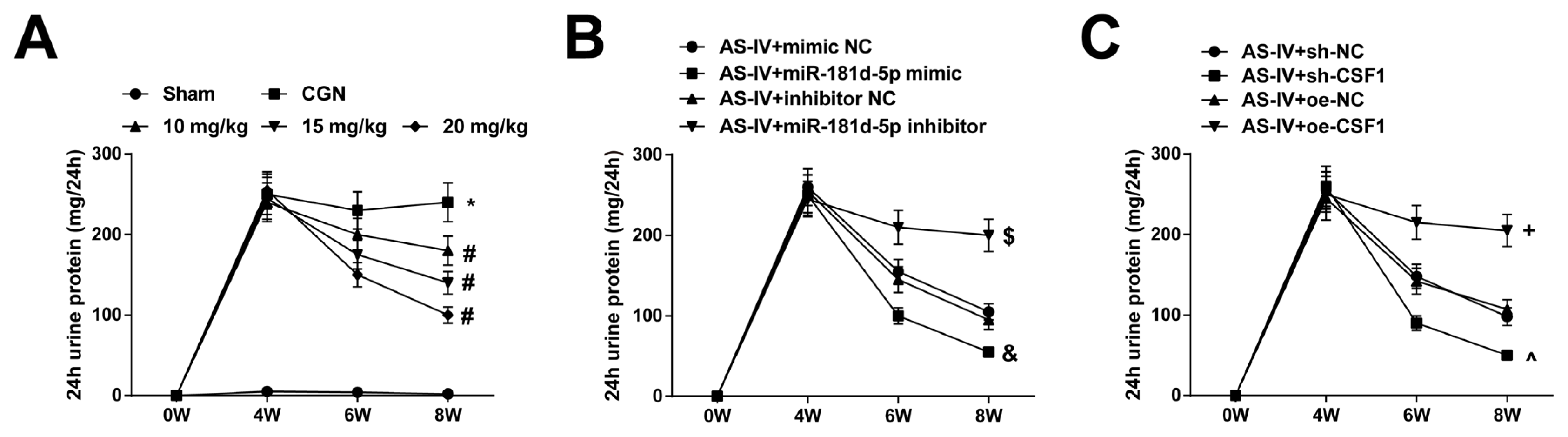

Supplement: Figure S1 — Detection of 24-h urinary protein in CGN rats. A–C: 24 h urinary protein of rats; Values were expressed as mean ± standard deviation (n = 6). *p < 0.05 vs. sham; # p < 0.05 vs. CGN; & p < 0.05 vs. AS-IV + mimic NC; $ p < 0.05 vs. AS-IV + inhibitor NC; ^ p < 0.05 vs. AS-IV + sh-NC; ^ p < 0.05 vs. AS-IV + oe-NC. [file turkjbiol-47-1-61s1.tif]
